# Chronic pain and post-traumatic stress in older patients with psychiatric disorders during the Covid-19 pandemic: co-occurrence and influence of attachment and personality factors

**DOI:** 10.1186/s12877-025-06422-6

**Published:** 2025-10-23

**Authors:** Hélène Saint-Martin, Jean-Michel Dorey, Mathieu Herrmann, Bernard Laurent, Cécile Lebrun-Givois, Catherine Perrot, Arlette Edjolo, Elisabeth Ouss, Elodie Pongan, Isabelle Rouch

**Affiliations:** 1 Department of Aging Psychiatry, Le Vinatier Hospital Center, 95 Boulevard Pinel, Bron, 69500 France; 2 INSERM, U1219, ACTIVE Team, Bordeaux Population Health Center, University of Bordeaux, Bordeaux, France; 3 Memory Clinical and Research Center of Saint-Etienne, University Hospital of Saint-Etienne, Saint-Etienne, France; 4 INSERM, U1028, CNRS, UMR 5292, Neuropain Team, Lyon Neuroscience Research Center, Lyon, France; 5 Department of Child and Adolescent Psychiatry, APHP Necker Hospital, Paris, France; 6 Memory Clinical and Research Center of Lyon, Hospices Civils de Lyon, Charpennes Hospital, Villeurbanne, France

**Keywords:** older age, chronic pain, post-traumatic stress disorder, ptsd, covid-19, attachment, personality

## Abstract

**Objectives:**

The Covid-19 pandemic context may have had numerous effects on the health of older patients with psychiatric disorders (PD), confronting them with a new source of stress and hindering their access to care. The aim of this study was to assess the long-term effects of the pandemic on both chronic pain (CP) and post-traumatic stress (PTS); the comorbidity of the two disorders; and to identify common psychological risk factors.

**Design:**

Medical interviews were conducted during and after (12 and 18 months later) the first lockdown.

**Setting:**

The STERACOVID longitudinal cohort study, conducted in two French hospitals.

**Participants:**

71 patients aged 65 or over; treated in an outpatient psychiatric service; and free of major neurocognitive disorders.

**Measurements:**

Validated scales were used to assess CP (ICD-11 criteria); PTS (PCL-S); personality traits (BFI-Fr); attachment style (RSQ); and coping strategies (BRIEF-COPE). χ² and Student’s t-tests, analyses of variance and logistic regression were used to compare patients with or without CP and/or PTS, in terms of attachment styles, personality traits and coping strategies.

**Results:**

CP and PTS were frequent and often co-occurring at T2. Fearful and preoccupied attachment styles and neurotic and extraverted personality traits were associated with the development of these two disorders; while coping strategies were not determinant.

**Conclusions:**

Our study identified factors associated with a higher risk of developing CP and/or PTS in the pandemic context. Assessment of attachment style and personality traits in clinical routine could help identify patients who are most vulnerable to this type of stress, and prevent the development of disabling chronic conditions.

## Introduction

Chronic pain (CP) is a frequent condition in older people, as its prevalence increases with age – reaching up to 50% of the over-75s [1]. CP is also often associated with psychiatric conditions, in a bidirectional relationship: thus, people with CP have an increased risk of developing sleep disorders, anxiety or depression, or even suicidal thoughts [2, 3]; and people with depression or bipolar disorder are more likely to have CP [4, 5]. Even though studies on the subject remain scarce, we can therefore suppose a high prevalence of CP in older people with psychiatric disorders (PD).

This population may also be at risk for developing post-traumatic stress disorder (PTSD) in the face of a traumatic event – like the Covid-19 pandemic. Thus, 90% of the over-55s would have experienced at least one traumatic event in their life [6]; and the physical and socio-psychological resources needed to cope with stress may become more limited with age [7]. Furthermore, the rates of traumatic event exposures and PTSD respectively appear to be much higher in major PD samples than in community samples [8].

CP and PTSD share a common pathological mechanism. While acute pain and stress act as necessary adaptive responses for survival, their chronicity proves inappropriate. Indeed, the physical manifestations of stress and the sensation of pain act as alarm systems to warn the individual of a physical or psychological threat, activate the response systems to this danger, and anticipate future similar threats. But if these physiological manifestations are registered continuously, their alarm function becomes obsolete and the individual spends energy responding to a threat that is no longer present or against which they can do nothing – making them more vulnerable. In fact, numerous studies have highlighted the frequent co-occurrence of CP and PTSD: a recent review conducted by Brennstuhl et al. [9] from 2000 to 2015 found a PTSD prevalence of 7.3–46% among CP sufferers, depending on pain syndromes and studies; and conversely, 45–66% of people with PTSD could present with CP.

Several models have emerged to explain this co-occurrence [9–12]. Among these, Asmundson et al. [10] hypothesize that CP and PTSD share a common vulnerability etiology: anxiety sensitivity, defined as a dispositional tendency to fear anxious symptoms, linked to the belief that these symptoms constitute a life-threatening risk.

Among these different determinants for CP and PTSD, literature highlighted the predominant role of certain early psychological factors, in particular attachment and personality. These factors are indeed known to predict certain behaviors - in particular health behaviors - and could then orient health trajectories throughout life. Regarding attachment, many authors have demonstrated its role in the development and maintenance of CP and PTSD - as established respectively in Meredith et al.’s [13] and Nohales et al.’s [14] reviews. The former demonstrate that insecure attachment could be a vulnerability factor to pain-related stress and favor pain chronicization. The latter highlight the interdependent relationship between insecure attachment (particularly the preoccupied subtype) and PTSD. Regarding personality, there are overlaps between traits identified as predictors of the development of CP on the one hand [15] and PTSD on the other [16], in particular harm avoidance and neuroticism. Furthermore, patients with CP or PTSD are characterized by a high prevalence of personality disorders. Thus, 31–59% of CP patients would present this type of disorders [17], and up to 24% of patients with PTSD would meet the diagnostic criteria for a borderline personality disorder [18].

Studies examining the impact of these early factors (attachment and personality) on both CP and PTSD remain, however, very scarce; and the diversity of models and tools used to assess these factors limits the comparability of studies on these two disorders respectively. Therefore, explanatory models for the link between CP and PTSD still lack evidence [19]. Furthermore, they have not been tested in older and/or PD populations.

In addition, numerous studies have demonstrated the impact of the Covid-19 pandemic on psychological health across different populations, with an increase in depressive and anxious symptoms [20, 21], fatigue, loneliness and poor well-being [22, 23] or PTSD symptoms [24] (for reviews, see [25, 26]). A rapid review of the impact of the pandemic on older adults [27] highlighted the need for studies on specific consequences and needs of more at-risk older adults – which is the purpose of the STERACOVID study, from which our data originate.

To fill these gaps, this study was aimed to search for an eventual association between CP and PTSD in older patients with PD, and to identify factors associated with the occurrence or maintenance of both disorders in the pandemic context.

## Methods

### Study design

This analysis was based on data from the STERACOVID study, a longitudinal study including a sample of older patients with PD, and followed up by teleconsultation during (T0) and after (T1, 12 months later; T2, 18 months later) the first lockdown related to the Covid-19 pandemic. The protocol has been published [28].

### Study sample

Seventy-one patients were included. In accordance with the inclusion and exclusion criteria of the STERACOVID study [28] they were all aged 60 or over; regularly followed in an outpatient unit of the department of aging psychiatry of Le Vinatier Hospital Center (Lyon, Bron) or the University Hospital of Saint-Etienne for a psychiatric pathology; affiliated or entitled to a social security scheme; free of major neurocognitive disorders; and not hospitalized at time of inclusion.

### Data collection

The interviews were conducted by telephone.

The first interview (T1) was divided into three parts. The first part collected, via closed questions and ordinal scales, socio-demographic data; lockdown conditions; psychiatric symptoms; and pain symptoms. The second and third parts assessed, via standardized scales, mental health status (anxiety, depression); coping strategies for the health crisis (BRIEF-COPE); personality traits (BFI-Fr); attachment style (RSQ).

The first part of the second interview (T2) was similar. The second and third parts used standardized scales to assess mental health status (anxiety, depression, and PTS symptoms via PCL-S) and quality of life.

Data relating to the first lockdown (T0) were collected retrospectively from the patients’ medical records.

### Dependent variables

The presence of CP and its treatment at T1 and T2, and the level of post-traumatic stress symptoms (PTS) at T2 were the dependent variables.

CP was defined as any daily pain lasting for more than three months, in accordance with ICD-11 criteria (Treede et al., 2015). These criteria were validated using two questions: “At present, do you suffer every day from one or more pain(s)?” and, if so, “How long have you suffered from it/them?“. Current analgesic treatment was recorded.

The PTSD Checklist Scale (PCL-S) [30] assessed the presence of PTSD symptoms in relation to the pandemic situation, using 17 items (e.g., “How much have you been bothered by feeling jumpy or easily startled in the past month?”) on a 5-point Likert scale (ranging from “Not at all” to “Extremely”). The level of PTS symptoms (i.e., PTS) corresponded to the PCL-S total score. The presence of a PTS syndrome (i.e., PTSS) was defined by a PCL-S-score above 31, as recommended for populations of all ages including women [31]. This cut-off score was chosen with regard to the absence of age-specific threshold validity in this population, and the particularly high prevalence rate (60.56%) obtained with the cut-off score of 24 suggested by Yeager and Magruder [32] for screening for PTSD in the over-65s.

### Explanatory variables

Age in years, gender and educational level (i.e. whether or not the patient had obtained a baccalaureate) were collected at T0. Covid-19 infectious status was collected at T0 and T1.

The French version of the Big Five Inventory (BFI-Fr) [33] was used to assess personality according to the Big Five model [34]. This scale includes 45 items (e.g., “I see myself as someone who is reserved”), rated on a 5-point Likert scale (ranging from “Disagree strongly” to “Agree strongly”). Each personality factor (extraversion; agreeableness; conscientiousness; neuroticism; openness) is assigned a score, corresponding to the average of the items assessing that factor (eight to 10 items per factor).

**Fig. 1:**
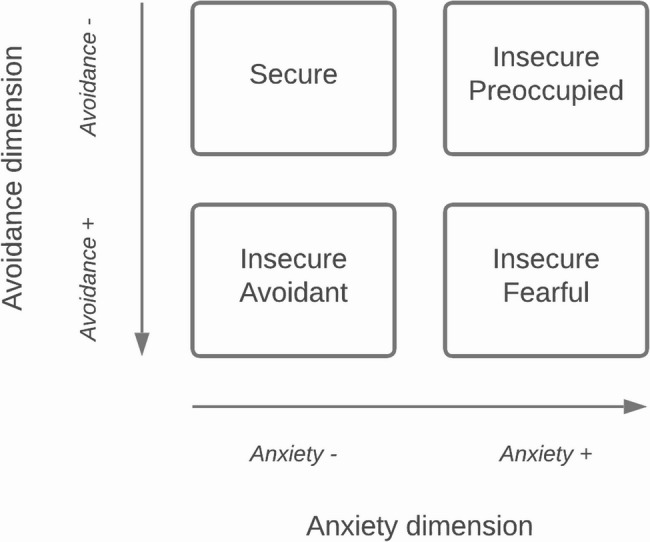
Two-dimensions attachment style model, based on Brennan et al. (1998)

The French version of the Relationship Scales Questionnaire (RSQ) [35] was used to assess attachment style according to a two dimensions/four styles model [36–38] (Fig. 1). This model stems from research into adult attachment styles based on the theory of Bowlby [39]. The intersection of the “self model” (avoidance/anxiety) and “other model” (dependence) dimensions gives rise to four styles of attachment to others, that can be assessed using self- and hetero-questionnaires. The RSQ includes 30 items (e.g., “I am comfortable depending on other people”), rated on a 5-point Likert scale (ranging from “Not at all like me” to “Very much like me”). Each attachment style (secure; avoidant; preoccupied; fearful) is assigned a score, corresponding to the average for the items assessing that attachment style (four to five items per style). Since the four styles are not represented in the same proportion within the population [37] and in order to obtain an attachment profile corresponding to the most prevalent style for each individual, we standardized the attachment scores obtained (i.e., centered reduced relative to the sample mean). Patients were then separated into four groups according to their highest z-score: S>(i.e., patients whose secure z-score is the highest of the four z-scores); A>; P>; F>. For further analyses, the groups were split in half according to either:

- The anxiety dimension of the model of attachment: the AnxietyDimension + class gathers P > and F > groups; the AnxietyDimension- class gathers S > and A > groups.
- The avoidance dimension: the AvoidanceDimension + class gathers A > and F > groups; the AvoidanceDimension- class gathers S > and P > groups.

The French version of the Brief Coping Orientation to Problems Experienced Inventory (BRIEF-COPE) [40] was used to assess strategies used by the individual to cope with pandemic-related stress. The scale includes 14 pairs of items (i.e., “I’ve been saying to myself “this isn’t real”” and “I’ve been refusing to believe that it has happened”), each rated on a 4-point Likert scale (ranging from “I haven’t been doing this at all” to “I’ve been doing this a lot”). Each pair is assigned a score corresponding to the sum of the scores of the two items. Each type of coping strategy (social support; problem-solving; avoidance; positive thinking) is assigned a score, corresponding to the average of the scores of the pairs assessing that type of strategy (two to five pairs of items per type) - as suggested by Baumstarck et al. [41].

DSM-5 criteria were used to characterize mood disorders [42]. Among the nine items of criterion A associated with major depressive episode (MDE), five had to be present, including at least one of the first two criteria, to validate the presence of a MDE.

### Statistical analyses

Continuous variables were described in means and standard deviations; categorical variables were expressed in percentages.

Different groups were defined according to the presence or absence of CP at T1 and T2: CP1 + CP2+ (i.e., presence of CP at T1 and T2); CP1 + CP2- (i.e., CP disappeared at T2); CP1-CP2+ (i.e., CP appeared at T2); CP1-CP2- (i.e., absence of CP at T1 and T2). As CP1 + CP2- comprised only one patient, analyses were made comparing only three groups: CP1+ (i.e., presence of CP at T1, gathering CP1 + CP2 + and CP1 + CP2-); CP1-CP2+; CP1-CP2-. Two groups of patients were then defined according to the presence (PTSS2+) or absence (PTSS2-) of PTSS at T2. Finally, four groups were defined according to the presence or absence of CP and PTSS at T2: CP2 + PTSS2+; CP2 + PTSS2-; CP2-PTSS2+; CP2-PTSS2-.

Comparisons were made using χ² tests for categorical variables, and Student’s t-tests or analyses of variance for continuous variables. Multinomial logistic regression analyses were then used to assess the effect of attachment styles and personality traits on CP and/or PTSS, adjusting for age, gender and educational level. A first model compared CP1+, CP1-CP2 + and CP1-CP2-, taking the latter as the reference group. A second model compared CP2 + PTSS2+, CP2 + PTSS2-, CP2 + PTSS2- and CP2-PTSS2-, taking the latter as the reference group. This division into four groups enables us to identify factors common to the occurrence of one, the other or both disorders jointly.

Test results were considered significant at the *p* <.05 threshold. Statistical analyses were performed using the 21 st version of SPSS software (SPSS Software, Chicago, USA).

## Results

The sample included 71 participants (55 women) aged 76.31 years (SD 7.07). Table 1 describes the participants’ characteristics.

**Table 1:** Characteristics of the study sample according to the pain status at T1 and T2, and the PTS status at T2

		All (*n* = 71)		CP1+ (*n* = 11)	CP1-CP2+ (*n* = 29)	CP1-CP2- (*n* = 31)	Statistic	*p*-value		PTSS2+ (*n* = 22)	PTSS2- (*n* = 49)	Statistic	*p*-value
Age, mean (SD)		76.31 (7.07)		81.64 (4.50)	75.90 (7.53)	74.81 (6.65)	F = 4.23	**0.019**		75.27 (8.44)	76.78 (6.41)	F = 0.68	0.412
Gender, frequency (%)							χ2 = 2.16	0.339				χ2 = 6.74 × 10^−4^	0.979
Women		55 (77.47%)		8 (72.73%)	25 (86.21%)	22 (70.97%)				17 (77.27%)	38 (77.55%)		
Men		16 (22.54%)		3 (27.27%)	4 (13.79%)	9 (29.03%)				5 (22.72%)	11 (22.45%)		
Educational level, frequency (%)							χ2 = 1.81	0.404				χ2 = 0.02	0.900
No baccalaureate		47 (66.20%)		9 (81.82%)	20 (68.97%)	18 (58.07%)				15 (68.18%)	32 (65.31%)		
Baccalaureate		23 (32.40%)		2 (18.18%)	9 (31.03%)	12 (38.71%)				7 (31.82%)	16 (32.65%)		
Infected to Covid-19, frequency (%)													
At T0		1 (1.41%)		1 (9.09%)	0 (0.00%)	0 (0.00%)	χ2 = 4.98	*0.083*		1 (4.55%)	0 (0.00%)	χ2 = 2.41	0.121
At T1		4 (5.63%)		1 (9.09%)	2 (7.14%)	1 (3.23%)	χ2 = 0.70	0.706		0 (0.00%)	4 (8.16%)	χ2 = 1.94	0.163
Major depressive episode, frequency (%)							χ2 = 1.43	0.489				χ2 = 1.52	0.217
No		63 (88.73%)		9 (81.82%)	25 (86.21%)	29 (93.55%)				18 (81.82%)	45 (91.84%)		
Yes		8 (11.27%)		2 (18.18%)	4 (13.79%)	2 (6.45%)				4 (18.82%)	4 (8.16%)		
RSQ score, mean (SD)													
RSQ-S – Secure		3.45 (0.63)		3.27 (0.61)	3.41 (0.56)	3.56 (0.70)	F = 0.91	0.406		3.28 (0.56)	3.53 (0.65)	F = 2.43	0.124
RSQ-A – Avoidant		3.69 (0.78)		3.67 (0.46)	3.69 (0.79)	3.70 (0.87)	F = 0.01	0.993		3.66 (0.88)	3.71 (0.73)	F = 0.05	0.833
RSQ-P – Preoccupied		2.25 (0.72)		2.24 (0.72)	2.25 (0.72)	2.25 (0.75)	F = 5.14 × 10^−4^	0.999		2.53 (0.67)	2.12 (0.72)	F = 5.16	**0.026**
RSQ-F – Fearful		3.06 (0.84)		3.23 (0.85)	3.22 (0.89)	2.85 (0.76)	F = 1.76	0.179		3.40 (0.80)	2.90 (0.81)	F = 5.66	**0.020**
RSQ profile, frequency (%)							χ2 = 6.84	0.336				χ2 = 6.05	0.109
S>		22 (30.99%)		4 (36.36%)	7 (24.14%)	11 (35.48%)				3 (13.64%)	19 (38.78%)		
A>		15 (21.13%)		1 (9.09%)	5 (17.24%)	9 (29.03%)				4 (18.18%)	11 (22.45%)		
P>		19 (26.76%)		2 (18.18%)	9 (31.03%)	8 (25.81%)				8 (36.36%)	11 (22.45%)		
F>		15 (21.13%)		4 (36.36%)	8 (27.59%)	3 (9.68%)				7 (31.82%)	8 (16.33%)		
BFI-Fr score, mean (SD)													
BFI-E – Extraversion		3.04 (0.80)		2.96 (0.82)	3.12 (0.84)	3.01 (0.79)	F = 0.21	0.810		2.80 (0.71)	3.16 (0.83)	F = 3.14	*0.081*
BFI-A - Agreeableness		4.25 (0.37)		4.22 (0.35)	4.23 (0.43)	4.27 (0.33)	F = 0.11	0.900		4.22 (0.38)	4.26 (0.37)	F = 0.12	0.736
BFI-C - Conscientiousness		3.82 (0.65)		3.98 (0.98)	3.79 (0.50)	3.79 (0.64)	F = 0.39	0.676		3.88 (0.88)	3.79 (0.52)	F = 0.26	0.611
BFI-N - Neuroticism		3.47 (0.80)		3.90 (0.33)	3.35 (0.92)	3.43 (0.77)	F = 2.02	0.141		3.81 (0.65)	3.31 (0.82)	F = 6.35	**0.014**
BFI-N - Openness		3.55 (1.21)		3.60 (0.59)	3.56 (0.63)	3.72 (1.70)	F = 0.67	0.516		3.31 (0.64)	3.66 (1.39)	F = 1.27	0.264
BRIEF-COPE score, mean (SD)													
COPE-SS – Social support		4.39 (1.52)		4.71 (1.72)	4.51 (1.44)	4.18 (1.54)	F = 0.62	0.540		4.57 (1.67)	4.32 (1.46)	F = 0.42	0.522
COPE-PS – Problem solving		4.71 (1.35)		4.32 (0.87)	4.66 (1.41)	4.90 (1.44)	F = 0.80	0.456		4.36 (1.23)	4.87 (1.39)	F = 2.14	0.148
COPE-A – Avoidance		3.94 (0.88)		3.64 (0.91)	4.05 (0.92)	3.94 (0.83)	F = 0.88	0.422		4.03 (0.95)	3.90 (0.85)	F = 0.33	0.570
COPE-PT – Positive thinking		4.30 (1.30)		4.33 (1.19)	4.28 (1.43)	4.30 (1.24)	F = 0.01	0.992		3.97 (1.08)	4.44 (1.37)	F = 2.06	0.156

### Prevalence of CP, PTSS and their co-occurrence

There was a significant increase in the prevalence of CP between T1 and T2 (χ2 (1) = 7.87, *p* =.005), from 16.90% at T1 to 54.93% at T2. The number of patients with analgesic treatment did not increase significantly between T1 (16.90%) and T2 (22.54%) (χ2 (1) = 3.03, *p* =.082).

Concerning PTS symptoms at T2, the mean score on the PCL-S was 28.79 (SD 8.69), and 30.99% of patients had PTSS.

Concerning the co-occurrence of CP and PTSS: the level of PTS and the prevalence of PTSS were significantly higher among CP2 + patients compared to CP2- (30.90 vs. 26.22, t (69) = −2.33, *p* =.023; 41.03% vs. 18.75%, χ2 (1) = 4.08, *p* =.043). There also was a significant difference between patients with CP at T1, those who had developed CP between T1 and T2 and those without pain (F (2, 68) = 4.03, *p* =.022): CP1 + patients had a mean score of 34.82 (SD 9.02) on the PCL-S; CP1-CP2 + had a score of 28.93 (SD 9.21); and CP1-CP2- had a score of 26.52 (SD 7.16).

### Risk factors

There was a significant effect of age on the evolution of CP between T1 and T2: post-hoc comparisons revealed that CP1 + patients were significantly older than CP1-CP2- patients (t (70) = −2.88, *p* =.015). There was no effect of Covid-19 infections, educational level or MDE diagnosis on CP or PTSS, nor for each coping strategy score (Tables 1 and 2).

**Table 2:** Characteristics of the study sample according to the pain and PTS status at T2

	CP2 + PTSS2+ (*n* = 16)	CP2 + PTSS2- (*n* = 23)	CP2-PTSS2+ (*n* = 6)	CP2-PTSS2- (*n* = 26)	Statistic	*p*-value
Age, mean (SD)	76.81 (8.93)	77.96 (6.79)	71.17 (5.64)	75.73 (6.54)	F = 1.60	0.198
Gender, frequency (%)					χ2 = 1.17	0.761
Women	13 (81.25%)	19 (82.61%)	4 (66.67%)	19 (73.08%)		
Men	3 (18.75%)	4 (17.39%)	2 (33.33%)	7 (26.92%)		
Educational level, frequency (%)					χ2 = 3.57	0.312
No baccalaureate	10 (62.50%)	18 (78.26%)	5 (83.33%)	14 (53.85%)		
Baccalaureate	6 (37.50%)	5 (21.74%)	1 (16.67%)	11 (42.31%)		
Infected to Covid-19, frequency (%)						
At T0	1 (6.25%)	0 (0.00%)	0 (0.00%)	0 (0.00%)	χ2 = 3.78	0.286
At T1	0 (0.00%)	3 (13.04%)	0 (0.00%)	1 (3.85%)	χ2 = 4.06	0.255
Major depressive episode, frequency (%)					χ2 = 2.58	0.462
No	13 (81.25%)	20 (86.96%)	5 (83.33%)	25 (96.15%)		
Yes	3 (18.75%)	3 (13.04%)	1 (16.67%)	1 (3.85%)		
RSQ score, mean (SD)						
RSQ-S – Secure	3.24 (0.50)	3.42 (0.56)	3.40 (0.75)	3.63 (0.71)	F = 1.38	0.256
RSQ-A – Avoidant	3.70 (0.76)	3.67 (0.70)	3.57 (1.23)	3.74 (0.77)	F = 0.09	0.967
RSQ-P – Preoccupied	2.46 (0.68)	2.16 (0.69)	2.72 (0.68)	2.09 (0.75)	F = 1.92	0.134
RSQ-F – Fearful	3.39 (0.84)	3.12 (0.90)	3.42 (0.77)	2.71 (0.69)	F = 2.97	**0.038**
RSQ profile, frequency (%)					χ2 = 10.61	0.304
S>	2 (12.50%)	8 (34.78%)	1 (16.67%)	11 (42.31%)		
A>	2 (12.50%)	4 (17.39%)	2 (33.33%)	7 (26.92%)		
P>	6 (37.50%)	5 (21.74%)	2 (33.33%)	6 (23.08%)		
F>	6 (37.50%)	6 (26.09%)	1 (16.67%)	2 (7.69%)		
BFI-Fr score, mean (SD)						
BFI-E – Extraversion	2.73 (0.68)	3.29 (0.87)	2.98 (0.81)	3.04 (0.79)	F = 1.59	0.200
BFI-A - Agreeableness	4.22 (0.43)	4.21 (0.38)	4.23 (0.21)	4.30 (0.36)	F = 0.26	0.854
BFI-C - Conscientiousness	3.93 (0.88)	3.77 (0.47)	3.74 (0.95)	3.81 (0.56)	F = 0.22	0.882
BFI-N - Neuroticism	3.77 (0.58)	3.38 (0.89)	3.92 (0.87)	3.26 (0.76)	F = 2.22	*0.094*
BFI-N - Openness	3.36 (0.61)	3.47 (0.65)	3.18 (0.78)	3.83 (1.81)	F = 0.81	0.491
BRIEF-COPE score, mean (SD)						
COPE-SS – Social support	4.53 (1.60)	4.55 (1.49)	4.67 (1.99)	4.11 (1.43)	F = 0.50	0.687
COPE-PS – Problem solving	4.22 (1.03)	4.83 (1.42)	4.75 (1.70)	4.90 (1.39)	F = 0.94	0.429
COPE-A – Avoidance	3.85 (0.82)	4.04 (1.00)	4.50 (1.17)	3.78 (0.69)	F = 1.27	0.291
COPE-PT – Positive thinking	4.08 (1.08)	4.38 (1.53)	3.67 (1.14)	4.50 (1.23)	F = 0.86	0.467

Multinomial logistic regression analysis (Table 3) found a link between fearful attachment style and extraverted personality trait on CP. CP1 + patients were significantly more extraverted than CP1-CP2- patients. CP1-CP2 + patients were significantly more fearful than CP1-CP2- patients.

**Table 3:** Multinomial logistic regression showing sociodemographic factors and attachment and personality factors

	CP1+				CP1-CP2+		
	OR	p-value	95%CI		OR	p-value	95%CI
Age	1.23	**0.005**	1.07–1.43		1.02	0.717	0.93–1.10
Gender							
Women	1.01	0.995	0.16–6.24		3.83	*0.080*	0.85–17.23
Men	Ref				Ref		
Educational level							
No baccalaureate	10.72	**0.035**	1.17–97.83		1.04	0.953	0.30–3.64
Baccalaureate	Ref				Ref		
RSQ score							
RSQ-S – Secure	0.36	0.210	0.08–1.77		0.64	0.434	0.21–1.94
RSQ-A – Avoidant	0.64	0.486	0.18–2.25		0.85	0.696	0.37–1.96
RSQ-P – Preoccupied	0.38	0.154	0.10–1.45		0.79	0.583	0.34–1.85
RSQ-F – Fearful	4.87	*0.052*	0.99–24.04		2.71	**0.046**	1.02–7.20
BFI-Fr score							
BFI-E – Extraversion	3.61	**0.050**	1.00–13.04		1.66	0.282	0.66–4.19
BFI-A - Agreeableness	3.20	0.403	0.21–48.96		1.37	0.739	0.22–8.53
BFI-C - Conscientiousness	1.02	0.970	0.29–3.65		0.88	0.817	0.29–2.66
BFI-N - Neuroticism	2.94	0.107	0.79–10.87		1.09	0.837	0.47–2.56
BFI-N - Openness	0.90	0.859	0.27–3.03		0.80	0.641	0.32–2.03

The reference modality is: CP1-CP2-

PTSS2 + patients showed significantly higher levels of neuroticism, fearful attachment style and preoccupied attachment style than PTSS2- patients (Table 1).

Table 2 describes the characteristics of the sample according to the presence or absence of CP and/or PTSS at T2. There was a significant difference in the level of fearful attachment style between CP2 + PTSS2+; CP2 + PTSS2-; CP2-PTSS2+, and CP2-PTSS2- patients. In particular, post-hoc comparisons showed that patients with both disorders at T2 had a higher score than CP2-PTSS2- patients (t (70) = −2.66, *p* =.047). Multinomial logistic regression analysis (Table 4) showed an effect of fearful attachment style and extraverted personality traits. CP2 + PTSS2 + patients had a significantly higher fearful attachment score than CP2-PTSS2- ones. CP2 + PTSS2- patients were significantly more fearful and more extraverted than CP2-PTSS2- ones.

**Table 4:** Multinomial logistic regression showing sociodemographic factors and attachment and personality factors

	CP2 + PTSS2+				CP2 + PTSS2-				CP2-PTSS2+		
	OR	p-value	95%CI		OR	p-value	95%CI		OR	p-value	95%CI
Age	1.01	0.841	0.91–1.12		1.02	0.710	0.92–1.13		0.89	0.201	0.74–1.07
Gender											
Women	2.91	0.280	0.42–20.26		1.61	0.581	0.30–8.69		0.56	0.697	0.03–10.04
Men	Ref				Ref				Ref		
Educational level											
No baccalaureate	0.80	0.784	0.16–4.03		4.03	*0.093*	0.79–20.56		7.76	0.242	0.25–239.80
Baccalaureate	Ref				Ref				Ref		
RSQ score											
RSQ-S – Secure	0.52	0.392	0.12–2.31		0.65	0.549	0.16–2.64		0.95	0.965	0.08–10.91
RSQ-A – Avoidant	0.55	0.363	0.15–2.01		0.54	0.268	0.18–1.61		0.28	0.132	0.05–1.48
RSQ-P – Preoccupied	1.06	0.918	0.33–3.46		0.55	0.272	0.19–1.60		1.34	0.759	0.21–8.76
RSQ-F – Fearful	5.63	**0.029**	1.20–26.53		5.08	**0.017**	1.33–19.33		8.51	*0.056*	0.94–76.80
BFI-Fr score											
BFI-E – Extraversion	1.19	0.810	0.29–4.94		4.75	**0.019**	1.29–17.51		7.18	0.121	0.59–86.87
BFI-A - Agreeableness	3.80	0.351	0.23–62.81		1.65	0.650	0.19–14.40		2.61	0.640	0.05–146.84
BFI-C - Conscientiousness	1.57	0.486	0.44–5.56		0.77	0.728	0.17–3.42		0.54	0.663	0.04–8.39
BFI-N - Neuroticism	3.00	0.124	0.74–12.22		2.12	0.185	0.70–6.46		5.80	*0.052*	0.99–34.16
BFI-N - Openness	0.82	0.774	0.20–3.28		0.76	0.652	0.22–2.55		0.55	0.537	0.08–3.69

The reference modality is: CP2-PTSS2-

Analysis after standardization of scores and allocation into four groups according to attachment style (S>; A>; P>; F>) revealed no significant difference between groups, either in terms of prevalence of CP at T1 (χ2 (3) = 3. 22, *p* =.359) or at T2 (χ2 (3) = 6.02, *p* =.110); nor in the level of PTS (F (3, 67) = 2.05, *p* =.116) or PTSS prevalence; nor in the co-occurrence between the two disorders. Nevertheless, there was a significant difference between the two classes of the anxiety dimension of attachment: compared to patients with a secure or avoidant attachment profile (i.e., AnxietyDimension-), patients with a preoccupied or fearful profile (i.e., AnxietyDimension+) were significantly more likely to develop CP (67.64% vs. 43.24% of S>/A > patients, χ2 (1) = 4.26, *p* =.039) or PTSS (44.12% vs. 18.92% of S>/A > patients, χ2 (1) = 5.26, *p* =.022) at T2. The same analyses according to the avoidance dimension revealed no effect on the development of CP (χ2 (1) = 0.28, *p* =.0.60), PTSS (χ2 (1) = 0.78, *p* =.376) or both disorders jointly (χ2 (3) = 1.18, *p* =.759).

## Discussion

The aim of our study was to investigate the co-occurrence of CP and PTSS in older patients with PD, in the context of stress related to the Covid-19 pandemic; and to identify the factors determining the development of one, the other of both disorders. As hypothesized, we showed that CP and PTSS were frequently co-occurring; moreover, fearful and preoccupied attachment style as well as neurotic and extraverted personality traits were associated with the development of these two disorders, while coping strategies were not found to be determinant.

### CP and PTSD during the Covid-19 pandemic

We showed that CP highly increased during the Covid-19 pandemic, in line with other studies conducted during this period [43, 44]. Among the factors contributing to pain exacerbation, Yoshida identified the presence of PD and under-medication. Here, we could envisage some of the CP appeared between T1 and T2 to be linked to the latter aspect: patients who reported new pain could have thus seen it become chronic due to a lack of management, in a context of sanitary restrictions hampering continuity of care. Indeed, in parallel with the sharp rise in the number of patients with CP, there was only a slight increase in the number of those on analgesic treatment in our study.

With regard to PTSS: many patients reported PTS, and one-third of our sample presented PTSS linked to Covid-19 pandemic. This figure seems comparable to the prevalence rates found in the other few studies carried out during the same period [24]; and higher than those usually found in the over-65s, estimated at 2.5–3.1% [45, 46]. This discrepancy seems to confirm the traumatic nature of the pandemic context in older patients – comparably to younger adults [24] – and apart from any effect of Covid-19 infections, which had no impact on PTSD in our study.

### CP and PTSD co-occurrence

Our study confirms the frequent co-occurrence between CP and PTS highlighted in literature [9]: thus, 22.46% of our sample would meet the criteria for both disorders 18 months after the first lockdown. In addition, the prevalence of PTSS and the level of PTS symptoms were significantly higher among CP patients than pain-free ones – and all the more so as the pain syndrome lasts. Thus, patients who already had CP 12 months after the first lockdown had more PTS symptoms six months later than those who had developed CP in the meantime.

To our knowledge, only two studies have looked at the comorbidities between CP and PTSD during the Covid-19 pandemic. The first one [47] focused specifically on older patients with PD, and revealed a significant correlation between the level of PTS symptoms and the level of pain – without, however, addressing the chronic nature of pain. The second one [48] examined the evolution of CP-related disability and PTS symptoms in U.S. patients who presented CP and/or PTSD before the pandemic. Two months after the start of the pandemic, the authors found a reduction in CP-related disability in patients with CP; and a reduction in PTS symptoms in patients with PTSD; while this decrease was less marked in patients with both disorders. While this study had the advantage of including pre-pandemic data, it did not focus specifically on older patients and did not include longitudinal follow-up.

### Risk factors

Certain attachment and personality factors have been shown to contribute to the development of both CP and PTSS, and could explain their co-occurrence.

With regard to attachment, patients with both disorders and those with CP alone appear to be significantly more fearful than patients with no disorder at T2; while patients with PTSS appear to be more preoccupied than those with no PTSS at T2. These results are consistent with literature, showing an association between insecure attachment and CP [13] or PTS symptoms [14]. Insecure attachment is indeed associated with dysregulation of the physiological response to stress [49]: it represents a factor of vulnerability to stressors, and could prevent the care adherence and therapeutic alliance necessary to treat the physiological manifestations of stress - thus favoring their chronicity [13]. Nevertheless, among the different insecure attachment profiles, it is the preoccupied subtype rather than the fearful one that had been most regularly highlighted as favoring CP [13] and PTSD [14]. The existence of different attachment models, in three or four categories, limits the comparability between studies; but a dimensional analysis (Fig. 1) allows us to avoid some of the pitfalls associated with this lack of consensus. Thus, we showed that the “anxiety” or “dependence” dimension [36–38] predicted both the development of CP and PTSS: anxious attachment profiles (preoccupied and fearful subtypes) are significantly more likely to develop either of these two disorders than non-anxious ones (avoidant or secure subtypes). These results are in line with McWilliams and Bailey [50], who found an association between high levels of anxiety/dependence and various health problems - including CP. Regarding PTSD, Nohales et al. [14] showed that preoccupied-subtype profiles are characterized by an over-activation of the attachment system and a focus on the feeling of threat and distress – explaining the higher reporting of PTS than avoidant-subtype people, who would tend to keep emotions at bay and minimize the feeling of distress felt.

Regarding personality, a higher level of extraversion was found in CP patients without PTSS, in comparison to patients with PTSS or patients with neither of the two disorders. While these results may seem surprising at first glance, they appear to be consistent with other studies [51, 52]. One possible explanation lies in the fact that extraverted people, who are particularly attracted by social environments in which drinking, sexual and reckless driving behaviors are approved, may develop a riskier lifestyle [53]. They are thus more likely to have accidents [54, 55], increasing the risk of developing CP. Extraverted individuals also tend to express their emotions more readily and may report higher level of pain in experimental situations [56] as in daily life [57]. Secondly, we showed that neuroticism influences the development of PTSS, consistently with literature [58]. Nevertheless, the influence of neuroticism on CP and PTSD jointly was only tendential (OR 2.98; 95% CI 0.92–9.64). In view of these results, it would seem interesting to work on the influence of combinations between different personality factors; or even to use more complex personality models, for a better understanding of the variations within each personality type – especially neuroticism. For example, Kern and Friedman [53] suggest that patients with a high level of both neuroticism and conscientiousness may be less prone to the development of various health disorders, as they tend to be more health-conscious. On a different level, DeYoung’s 10-aspects model [59] suggests that neuroticism comprises a “volatility” subtype (or aspect), characterized by externalization of negative emotions and active defense against threats, and a “withdrawal” aspect, characterized by internalization of negative emotions and passive avoidance.

The co-occurrence of CP and PTSD could therefore be partially explained by attachment factors, and by personality factors to a lesser extent. The early development of these factors would support Asmundson et al.’s [10] assumption that CP and PTSD may share a common vulnerability etiology, residing in the “anxiety sensitivity” (AS) trait [60]. Interestingly, AS has been assumed to be – rather than a unitary trait – a composite set of cognitive biases, influenced by certain attachment factors or personality traits [61]. Thus, Cox et al. [62] demonstrated that neuroticism and extraversion predict AS levels; and Watt et al. [63] showed that insecurely attached individuals – specifically preoccupied and fearful profiles (i.e., AnxietyDimension+) – report significantly higher AS levels. Replicating Weems et al.’s [64] results, they found that, of the two dimensions model of attachment, the anxiety dimension accounted for unique variance in AS levels.

Conversely, coping mechanisms were not found to be influencing either of the two disorders, suggesting that systems rooted in early childhood, and in principle indelible, have a greater impact on health trajectories than cognitive coping mechanisms implemented in adulthood.

### Strengths and limitations

Our study is the first to examine the co-occurrence of CP and PTSD in older patients with PD during the Covid-19 pandemic. This context, although unfortunate, was particularly favorable for studying the impact of stress on health, as the entire population has experienced the same stress factor over a similar period of time. Among the clinical and public health implications of our observations: the high prevalence of CP and PTSD should prompt healthcare professionals to look for both disorders in a context of stress. The assessment of attachment style and personality traits in clinical routine could help to identify more accurately those who are most vulnerable to stress factors similar to the one we have experienced worldwide - in order to prevent the development of these particularly disabling conditions. Furthermore, regardless of the context, their frequent co-occurrence should prompt us not to overlook the presence of CP in the face of PTSD symptoms - and vice versa.

However, several limitations emerge from these results. First, the prevalence of CP at T1 seems surprisingly low in view of CP prevalence in our population. As this survey was carried out during the third lockdown in France, we can assume that some patients may have inhibited their pain experience and under-reported their pain, through a cognitive avoidance mechanism - the only strategy available to them at the time, as the health crisis prevented continuity of care. Pain symptoms could also have improved in this context, in a population particularly vulnerable to Covid-19. Zambelli et al. [65] found similar results, and speculated that the sense of security resulting from sanitary measures, as well as the reduction in strenuous, pain-inducing activities, may be responsible. The significant increase in CP between the two periods could therefore reflect both the exhaustion of patients’ resilience, in a stressful context that lasts and dries up their internal resources; and the reaction to a new stress, linked to the lifting of restrictions.

With regard to the assessment of PTSD, the absence of pre-pandemic data constitutes an important limitation, and does not allow us to ensure the absence of PTSD beforehand. However, the use of the situational version of the PCL-S theoretically enabled us to assess only health-crisis-related PTS, and not those linked to older stressors. Furthermore, PTSD-prevalence studies among older people remain scarce, and the low rates found by Kessler et al. [45] could be largely underestimated [66]. Thus, methods for assessing PTSD in this population may require adjustments – as suggested by Yeager and Magruder [32]. Validation studies of the PCL-5 (i.e., a DSM-5-based version of the PCL) among older people would be valuable in generating a consensus on the use of PTSD-assessing tools in this population.

Finally, with regard to our population, the sample size was relatively small, which limited the statistical power of the study. But this is the first longitudinal study to assess the influence of psychological factors on CP and PTS in a specific population of older patients with PD. It is important to consider this population at risk of psychiatric and physical decompensation. In addition, the proportion of women seems higher (77.46%) than in the French population (60.15%). As CP [1] and PTSD [14] were found to be more frequent in women than in men, we can assume that the prevalence of these two disorders may have been slightly overestimated.

## Data Availability

The datasets used and/or analyzed during the current study available from the corresponding author on reasonable request.
